# A Plea for Cæsarian Section

**Published:** 1919-03-15

**Authors:** 


### A PLEA FOR CÆSARIAN SECTION.

### Its Popularity and Justification.

There is no doubt that the modern trend of thought inclines more and more towards the employment of Cæsarian section, as the best treatment of many obstetric complications. Many of the prejudices which formerly assailed the popularity of this operation have now been removed. Improvements in modern operative technique, asepsis, and the perfection of surgical anæsthesia, have brought about this change. The operation now performed is a speedy and a safe one: the mortality, as regards the mother, being well below 2 per cent. In the experience of one obstetric surgeon, with a record of one hundred and twenty cases, the mortality was actually below 5 per cent. The infant mortality in most cases is practically negligible. So that in skilled hands, or even in the hands of a surgeon with moderate experience, the results of Cæsarian section compare very well with those of normal physiological labour, and are immeasurably superior to other methods of evacuating the full term pregnant uterus. Not that we suggest for one moment that Cæsarian section is a substitute for the normal physiological train of events which we term parturition, but that the operation should be increasingly used in many cases of difficult labour. We may mention, in passing, that the operation is becoming increasingly popular in other parts of the world, particularly in the United States of America, France, and Italy.

For the benefit of some of our readers we may briefly state that Cæsarian section is usually performed by the abdominal route, and that the uterus once exposed is opened by an incision in its upper portion: the child and "after-birth" are speedily delivered, the uterus and abdominal wall reconstituted with sutures, and the patient returned to bed.

### The Medical Aspect.

The chief medical indications for this operation are in cases of Placenta prævia, Eclampsia, Concealed ante-partim hemorrhage, and cases of obstruction to labour caused by smallness and deformity of the pelvis, or else obstruction to the normal fœtal exit, by some pathological condition, such as an ovarian dermoid cyst in the "Pouch of Douglas" below the pelvic brim. In cases of Eclampsia, the repute of the operation is now well established.

In this condition of Eclampsia the mother suffers from a succession of "fits," which in many cases would lead to a fatal termination if the uterus were not speedily evacuated. Quite recently, we examined a woman at full term who, when seen, had already suffered many eclamptic fits, she was comatose, and her prognosis, if left alone, seemed quite hopeless. The patient was speedily transferred to hospital, Cæsarian section performed, living twins delivered, and all three were discharged from the hospital three weeks after admission. The present high note of maternal mortality from Eclampsia would be much diminished if Cæsarian section were performed earlier in the disease. The great tendency is to leave these cases too long without operative interference.

In concealed ante-partim hemorrhage of any magnitude the condition is a desperate one, and many cases will perish in spite of any treatment: however, in recorded cases, recovery has resulted from an immediate Cæsarian section. Placenta prævia means that the placenta, or "after-birth," is situated on the floor instead of the roof of the uterine cavity: thus with the initiation of labour there will be hemorrhage as the mobile lower uterus separates itself from the immobile placenta.

Such hemorrhage results in very great fœtal and a greater maternal mortality. The great alternatives to Cæsarian section in treatment are the use of an often inadequate and septic water bag, or else the plugging of the gaping maternal blood sinuses by the breech of the fœtus—surely the most expensive "plug" in the whole world, as the proceeding, though saving the mother's life, is so often marred by fœtal death. If Cæsarian section be performed at the judicious time, that is, when the child is viable and the mother near term, a healthy mother and a living child will be the reward.

When there is an undue disparity between the size of the fœtal presenting part and the maternal osseus pelvic girdle, there are several alternative modes of procedure for the obstetrician to choose, but only one method by which success is assured, and that is Cæsarian section. In this regard one must consider the question of induction of labour with some respect, but although a capital method in theory, in practice a puny infant is often delivered, one which may with difficulty be reared, and in no way to be compared with the full-weight, healthy, Cæsarian baby. Other alternatives with which we have little or no sympathy are the undue use of obstetric forceps, or the application of destructive instruments such as the Cranioclast, or Cephalotribe. As a scientific proceeding instrumental delivery in cases of any difficulty is extremely crude. There is a definite use for obstetric forceps, and it consists of the guidance of the fœtal head through the maternal passages by means of gentle traction: but there is also the great abuse of ruthless manual force by which the child is literally hauled out of the genital tract. This procedure causes much shock to the mother, entails much bruising and laceration of maternal passages, and throws a quite unwarranted strain upon the pelvic articulations. Several cases of tubercle owe their incidence to this great strain so suddenly thrust upon a pelvic joint. Then again, the bladder may be damaged, and, perchance, the miseries of cystitis prolong the puerperium.

The fœtal head after a difficult forceps delivery is often much misshapen, and even though the child may live, it is often delicate and may be the victim of that dread disease infantile paralysis. If only statistics were collected, many cases of epilepsy, idiocy, and weak cerebration would be

**A Plea for Cæsarian Section**—(*continued*).

traced to this cause. Perforation and fœtal destruction as a means of delivery are simply prostitutions of the obstetric art, the fruition of the long days of gestation being destroyed, to the chagrin of the mother and to the detriment of the State.

BENEFIT TO THE MOTHER.

Cæsarian section is thought by many women to be the most painless of all modes of delivery. The mother, prior to operation, is given a sedative which will allay any fears she has, and may then be anæstheticised in her bed. The operation proceeds and she recovers consciousness later to find her babe safely born. Her own convalescence is a speedy one, and she should be able to walk about her house within three weeks of the operation. What a contrast to the miseries of a prolonged labour, culminating in the application of destructive instruments, and a puerperium marred by the grief of her absent babe, and perhaps serious injury to herself. We have already mentioned the low maternal mortality. Obstetric opinions differ as to the number of times Cæsarian section may with safety be performed, but the general concensus of opinion favours three or four operations.

CONCLUSIONS.

If the indications for Cæsarian section are recognised in good time—and there is no excuse for non-recognition—a healthy mother and a living child should be presented to the State. Early recognition of such indications can only be obtained from ante-natal visits for the rich and middle classes, and a scheme of ante-natal clinics for the great masses of the British people.

The want of such ante-natal clinics is urgent and of paramount importance.

Let the politicians stop their wrangles, let these ante-natal clinics be founded, and let us prevent an appalling waste of human life.

